# Sex-specific effect of *CPB2* Ala147Thr but not Thr325Ile variants on the risk of venous thrombosis: A comprehensive meta-analysis

**DOI:** 10.1371/journal.pone.0177768

**Published:** 2017-05-26

**Authors:** Nora Zwingerman, Alejandra Medina-Rivera, Irfahan Kassam, Michael D. Wilson, Pierre-Emmanuel Morange, David-Alexandre Trégouët, France Gagnon

**Affiliations:** 1 Division of Epidemiology, Dalla Lana School of Public Health, University of Toronto, Toronto, Canada; 2 Genetics and Genome Biology Program, SickKids Research Institute, Toronto, Canada; 3 Laboratorio Internacional de Investigación sobre el Genoma Humano, Universidad Nacional Autónoma de México, Juriquilla, Santiago de Querétaro, Querétaro, Mexico; 4 Department of Molecular Genetics, University of Toronto, Toronto, Canada; 5 Institut National de la Santé et de la Recherche Médicale (INSERM), Unité Mixte de Recherche (UMR) en Santé 1062, Nutrition Obesity and Risk of Thrombosis, Marseille, France; 6 Faculté de Médecine, Aix Marseille Université, Marseille, France; 7 Institut National de la Santé et de la Recherche Médicale (INSERM), Unité Mixte de Recherche en Santé (UMR_S) 1166, Paris, France; 8 Institute for Cardiometabolism and Nutrition, Paris, France; 9 Sorbonne Universités, Université Pierre et Marie Curie (UPMC Univ Paris 06), UMR_S 1166, Team Genomics & Pathophysiology of Cardiovascular Diseases, Paris, France; Institut d'Investigacions Biomediques de Barcelona, SPAIN

## Abstract

**Background:**

Thrombin activatable fibrinolysis inhibitor (TAFI), encoded by the Carboxypeptidase B2 gene (*CPB2*), is an inhibitor of fibrinolysis and plays a role in the pathogenesis of venous thrombosis. Experimental findings support a functional role of genetic variants in *CPB2*, while epidemiological studies have been unable to confirm associations with risk of venous thrombosis. Sex-specific effects could underlie the observed inconsistent associations between *CPB2* genetic variants and venous thrombosis.

**Methods:**

A comprehensive literature search was conducted for associations between Ala147Thr and Thr325Ile variants with venous thrombosis. Authors were contacted to provide sex-specific genotype counts from their studies. Combined and sex-specific random effects meta-analyses were used to estimate a pooled effect estimate for primary and secondary genetic models.

**Results:**

A total of 17 studies met the inclusion criteria. A sex-specific meta-analysis applying a dominant model supported a protective effect of Ala147Thr on venous thrombosis in females (OR = 0.81, 95%CI: 0.68,0.97; p = 0.018), but not in males (OR = 1.06, 95%CI:0.96–1.16; p = 0.263). The Thr325Ile did not show a sex-specific effect but showed variation in allele frequencies by geographic region. A subgroup analysis of studies in European countries showed decreased risk, with a recessive model (OR = 0.83, 95%CI:0.71–0.97, p = 0.021) for venous thrombosis.

**Conclusions:**

A comprehensive literature review, including unpublished data, provided greater statistical power for the analyses and decreased the likelihood of publication bias influencing the results. Sex-specific analyses explained apparent discrepancies across genetic studies of Ala147Thr and venous thrombosis. While, careful selection of genetic models based on population genetics, evolutionary and biological knowledge can increase power by decreasing the need to adjust for testing multiple models.

## Introduction

Cardiovascular diseases incidence, complications, response to treatment and burden vary between men and women [1, 2]. These outcome differences could be due to the sex-specific effects of cardiovascular disease risk factors, including hemostatic plasma protein levels, on disease expression [3]. Thrombin-activatable fibrinolysis inhibitor (TAFI) plasma levels, which play a critical role in maintaining the delicate balance between coagulation and fibrinolysis [4], are associated with thrombotic events [5–11]. TAFI levels have been shown to increase with age in females but not in males [6, 12, 13]. In addition of being partially explained by the effect of sex steroids [14], such sexual dimorphism could be due to natural selection impacting the genetic architecture of complex traits [15]. Sex can modify the effect of genetic variants. It has recently been shown that 12–15% of autosomal expression quantitative trait loci act in a sex-specific manner [16]. Specifically, genetic risk profiles have been shown to have sex-specific associations with cardiovascular disease [17]. A sex-specific effect was suggested in coronary heart disease for the Ala147Thr variant, in the *CPB2* gene encoding TAFI [17], but sex effect on venous thrombosis remains to be determined.

TAFI is a plasma protein, synthesized primarily by the liver, which circulates in plasma as an inactive precursor. TAFI activation occurs by trypsin-like enzymes such as thrombin, plasmin, and most efficiently by the thrombin-thrombomodulin complex on the vascular endothelial surface [18–20]. Active TAFI (TAFIa) down-regulates fibrinolysis by removing the carboxy-terminal lysine residues from partially degraded fibrin polymers during clot lysis, which prevents the binding and activation of plasminogen [21–23]. Through this mechanism, TAFIa stabilizes the fibrin clot making it more resistant to lysis and therefore decreases fibrinolytic activity [23–25]. The antifibrinolytic effect of TAFIa is dependent on TAFI plasma concentration, the rate of TAFI activation, and the stability of the active enzyme [18, 19, 26]. TAFI attenuates fibrinolysis through a threshold-dependent mechanism [27, 28], only when the TAFIa levels drop below the threshold does the rate of fibrinolysis increase exponentially.

The *CPB2* gene encodes TAFI. This gene is located on chromosome 13q14.11, spans approximately 48kb of genomic DNA that comprises 11 exons and 10 introns [29]. Variants in the *CPB2* gene have the potential to affect TAFI plasma levels, function, and stability and therefore may be functionally relevant to thrombotic disease risk. The two most studied *CPB2* variants are Ala147Thr and Thr325Ile, both resulting in amino acid substitutions. The anti-fibrinolytic effect of Ala147Thr variant in exon 6 does not appear to involve TAFI protein stability [26]. Interestingly variants encompassing exons 6–7 have been maintained by balancing selection [30], which may be a result of a heterozygote advantage, and one of the two major haplotype clades and the preferential exon splicing of exon 7 is associated mainly with one haplotype [30]. The Ile-325 variant in exon 10, results in increased TAFI protein stability [26] and its antifibrinolytic effects are 30–60% greater than the Thr-325 variant [26, 31]. As a result, homozygosity for Ile-325 would be predicted to result in a more potent enzyme and increase the risk of a thrombotic event. Conversely, Ile-325 homozygosity has been associated with decreased TAFI levels [31–33]. A possible explanation for the apparent inconsistency is that moderate changes in TAFI levels do not alter the function because of the threshold-dependent mechanism.

Despite the critical hemostatic role of TAFI, epidemiological studies on the effect of the above common TAFI-associated variants on the risk venous thrombosis are inconclusive [32, 34–55] with inconsistent results between studies, and a recent meta-analysis [56] that has important methodological limitations. We speculate that these inconsistencies could be due to the interaction of these variants with sex on the risk of thrombotic events, and to the lack of statistical power of individual studies. To simultaneously address these two points, we conducted a comprehensive systematic review and meta-analysis that examines whether these variants are associated with venous thrombotic events in a sex-specific manner.

## Materials and methods

### Search strategy

To identify studies that examined the association between variants of *CPB2* and thrombotic events, EMBASE, MEDLINE, HuGE Navigator literature, and the HuGE Navigator GWAS Integrator databases were searched from their inception through to September 2015 using a combination of keywords and MeSH terms, including “thrombosis”, “thromboembolism”, “pulmonary embolism”, “cerebral infarction”, “cerebral thrombosis” with “TAFI”, and “CPB2”. Titles and abstracts were screened by two independent reviewers, with records that reported the investigation of the *CPB2* variants and thrombotic events carried forward to full-text screening. Here, a thrombotic event was defined as a thrombus occurring in a vein, which may or not embolize and become a pulmonary embolism. Additional records were searched in the reference lists of records included in the full-text screening to achieve a comprehensive search.

During the full-text screening, two independent reviewers determined eligible studies using the following criteria:

- *Inclusion criteria*: Studies that investigated the association between thrombotic events and rs1926447 C/T (Thr325Ile), rs3742264 G/A (Ala147Thr), and/or *CPB2* variants in high (>0.8) linkage disequilibrium (LD) calculated for each ethnicity, including GWAS and other genetic association studies.
- *Exclusion criteria*: Review articles, genetic linkage studies, non-peer reviewed studies (e.g. conference proceedings).

Data was abstracted onto a standardized form by the two independent reviewers based on the Strengthening of Reporting of Genetic Association Studies (STREGA) [57] and Human Genome Epidemiology Network (HuGENet) [58] guidelines, which included (1) study details, including the title, author, country, year of publication, and SNP(s) being studied; (2) the research methods and study participants, including study design, mean age, ethnicity, proportion of females, definitions of case and non-case, and subject ascertainment; [59] genotype information, including source of DNA, method of genotyping, genotype distribution, genotype contrasts, and whether population stratification and Hardy-Weinberg Equilibrium (HWE) were addressed. When information was not available from the article, details were sought from papers reporting on the same study population, or by contacting respective authors. Each study was assessed for quality in accordance to STREGA [57] and HuGENet [58] guidelines. In particular, scores between zero and two were assigned each category in items (2) and [59] of the abstracted data, for a maximum score of 16. The list of quality assessment items is provided in S1 Table.

Finally, the corresponding authors of all included articles were contacted for sex-specific genotype counts for cases and non-cases.

### Meta-analysis

Crude odds ratios (OR) and 95% confidence intervals (CI) were calculated from the genotype frequencies for each study using the minor allele as the risk allele under dominant and recessive model for the Ala147Thr and Thr325Ile variants, respectively. These primary models for analysis were strategically selected *a priori* based on available population genetics, evolutionary and biological data. The dominant model (AA + GA vs. GG) was selected for the Ala147Thr variant based on both evolutionary and biological information: evidence of balancing selection [30], which could indicate a potential heterozygote advantage, and based on observation of increased mean TAFI Ag level for heterozygotes and homozygotes for the rare allele [32, 60]. The recessive model (TT vs. CT + CC) was selected for Thr325Ile variant based on evidence for homozygotes for the rare allele having changes in protein levels [31–33] and stability [26, 31]. An inverse-variance weighted random-effects model was then used to estimate a pooled effect estimate for venous thrombosis using Stata Version 12 software (StataCorp LP, College Station, Texas). The I^2^ statistic and Cochran’s *Q* statistic were used to assess the intensity and significance of between-study heterogeneity, respectively. Between-study heterogeneity was considered low, moderate, and high with corresponding I^2^ values of 25%, 50%, and 75% and a Cochran’s Q p-value <0.05 suggested statistically significant heterogeneity [61]. Funnel plots and Egger’s test were used to assess publication bias [62]. Sex-specific meta-analyses for each variant, including all studies with sex-specific genotype information, were performed; an interaction test was used to determine if the associations were significantly different between the sexes for each variant.

Potential sources of between-study heterogeneity were investigated by sensitivity analyses. First, since departure from HWE is a potential indicator of poor genotyping or ascertainment bias, studies that deviated from HWE were excluded. Departure from HWE was assessed in the non-cases using a chi-squared statistic with one degree of freedom. Second, studies were stratified by geographic region for a subgroup analysis to investigate the effects of potential population substructure. Third, studies with potential misclassification of case and non-case status were excluded (e.g. studies where the definition of a non-case encompassed individuals with other types of thrombotic events, for example a myocardial infarction; studies where the definition of cases included individuals with a medical history suggesting a predisposition to thrombotic events). Fourth, high-risk study populations (e.g. FVL mutation carriers, study samples with early disease onset) were analyzed separately to investigate the association for individuals who may already be genetically predisposed for a thrombotic event. Lastly, examination of whether the use of proxy SNPs in LD influenced the results.

While the primary analyses were performed using a genetic model that was pre-selected based on biological data of the variants, secondary analyses of the variants examined the association using alternative genetic models. The heterozygote effect, homozygote effect, dominant, recessive, and allelic models were tested for comparison with our hypothesized primary models, as if we would assume the wrong genetic model, statistical power would be decreased.

The cumulative evidence for association of the Ala147Thr and Thr325Ile variants was assessed according to the Venice guidelines [63] that use 3 major criteria: 1) amount of evidence; 2) replication of results; and 3) protection from bias.

## Results

**Fig 1:**
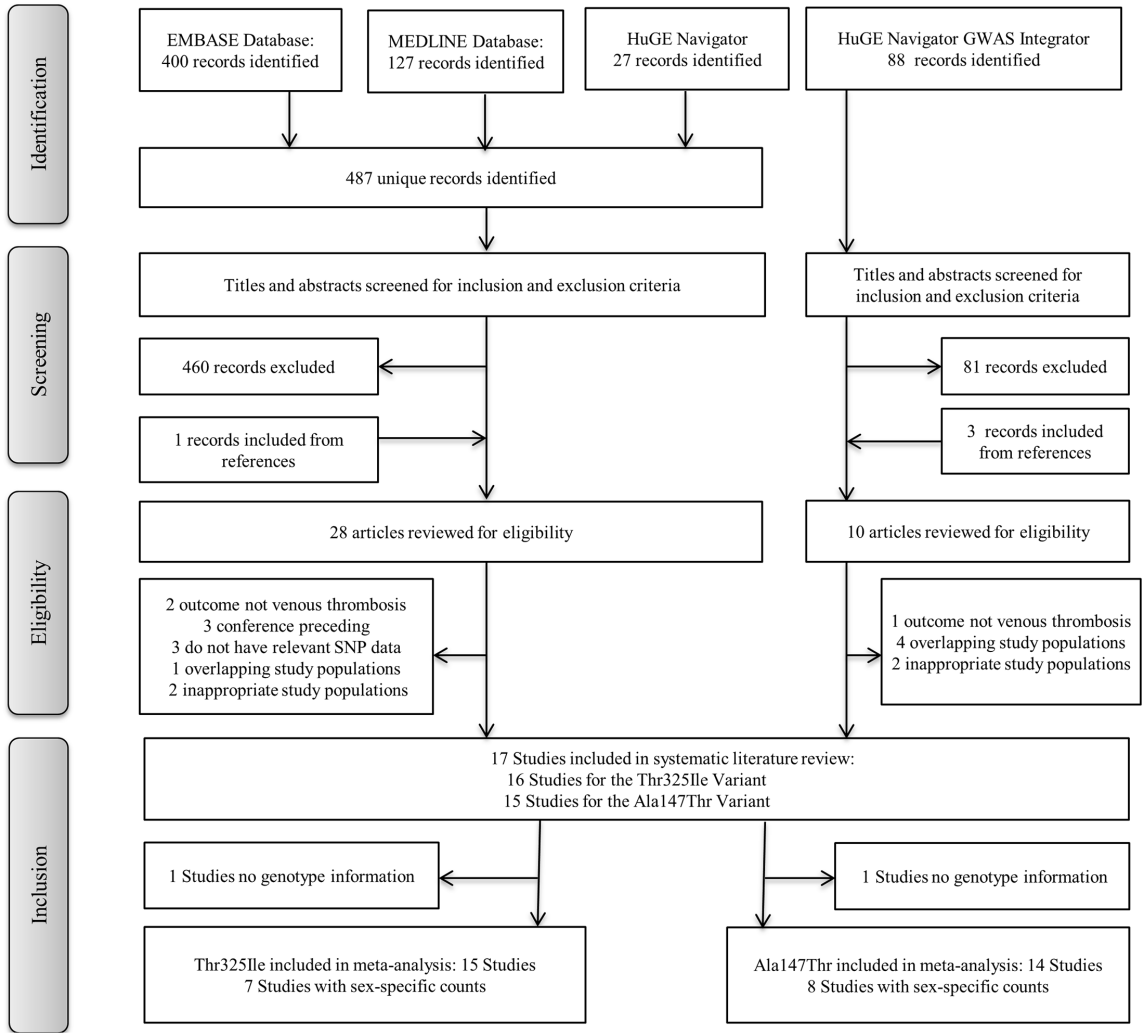
Flow-chart of the selection process for inclusion into the systematic review. Outline of the selection process used to identify studies from a comprehensive literature search that investigated the *CPB2* variants and venous thrombosis.

Overall 17 studies met the inclusion criteria and all met the quality assessment score to be included in this meta-analysis. For the Thr325Ile variant 15 studies had information and 14 studies had information for the Ala147Thr variant, with 12 studies containing information on both variants. A flow chart describing the selection process is presented in Fig 1, and a summary of the study characteristics is shown in S2 Table.

### Ala147Thr variant

In total, the meta-analysis for the *CPB2* Ala147Thr variant and venous thrombosis included a total of 8076 cases and 12 772 non-cases. The genotype distribution of Ala147Thr variant in case and non-case groups are shown in S3 Table. The minor allele frequency in non-cases varied from 24.5% in a French population [47] to 48.4% in Spanish population [51].

Under a dominant model, the odds of a venous thrombotic event is 0.94 (95% CI: 0.88, 1.02; p = 0.128) (S1 Fig). Overall, there was low heterogeneity (I^2^ = 16.8%, p = 0.270) across all studies, indicating consistent results across studies with a dominant model. There was an indication of small-study effects (p = 0.02) with the GWAS studies [50, 64, 65] having estimates closest to the null, however, the funnel plot appears fairly symmetrical with slightly more small studies on the left of the estimate (S2 Fig).

**Fig 2:**
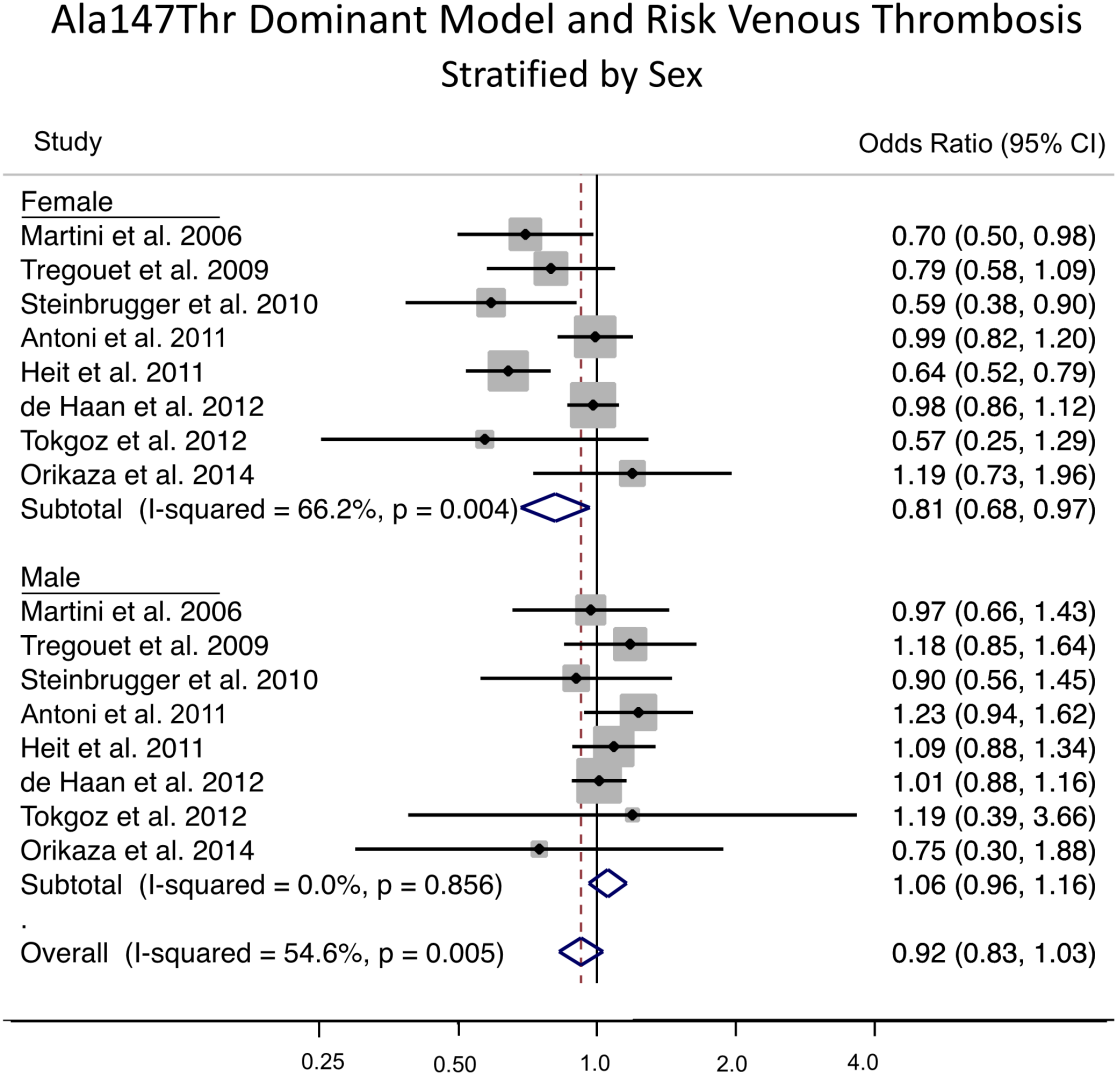
Sex-specific meta-analysis of the *CPB2* Ala147Thr variant using the dominant model and risk of venous thrombosis. The analysis was stratified by sex. The solid squares represent the ORs from the individual studies; horizontal lines represent corresponding CIs; the diamonds show the combined ORs.

The genotype distribution of Ala147Thr variant by sex in case and non-case groups is shown in Table 1. An examination of the allele frequencies by sex in non-cases showed a significantly higher minor allele frequency in females compared to males (p = 0.03) across all studies where genotypes counts was provided by sex. For the meta-analysis stratified by sex, for females 4058 cases and 5367 non-cases were analyzed and for males 3222 cases and 3989 non-cases were included in the analysis. Under a dominant genetic model (AA + GA vs. GG), subgroup analysis by sex for venous thrombosis showed an OR of 0.81 (95% CI: 0.68, 0.97; p = 0.018) from the eight studies with genotype frequencies in females and 1.06 (95% CI: 0.96, 1.16; p = 0.263) from the eight studies with genotype frequencies in males (Fig 2). The association differed by sex with a statistically significant interaction (p = 0.004). The forest plot shows that the OR estimate for five of the eight studies in females had estimates below 0.80. There was a moderate amount of between-study heterogeneity (I^2^ = 66.2%, p = 0.004) in females and no indication of between-study heterogeneity in males (I^2^ = 0%, p = 0.263). A potential source of heterogeneity is the combination of common forms of venous thrombosis (i.e. venous thromboembolism which is composed of deep vein thrombosis and pulmonary embolism) and less common forms of venous thrombosis (e.g. portal vein thrombosis, renal vein occlusion, cerebral venous infarction); however, this explanation is unlikely as stratified analysis showed the effect estimates in both groups to be consistent (results not shown). In the eight studies with sex-specific data, there was no indication of publication bias or small study effects (Egger’s test p = 0.392).

**Table 1:** Distribution of Ala147Thr genotypes by sex among cases and non-cases in studies included in the meta-analysis.

Included Study	GG Genotype		GA Genotype		AA Genotype		Minor Allele Frequency	
	Cases	Noncases	Cases	Noncases	Cases	Noncases	Cases	Noncases
MALES								
Martini et al. 2006	96	94	94	85	12	22	0.292	0.321
Tregouet et al. 2009	105	185	101	150	23	35	0.321	0.297
Steinbrugger et al. 2010	67	65	53	62	13	9	0.297	0.294
Antoni et al. 2011	234	174	238	143	50	31	0.324	0.295
Heit et al. 2012	332	322	323	305	90	63	0.338	0.312
de Hann et al. 2012	619	1020	584	925	120	222	0.311	0.316
Tokgoz et al. 2012	7	19	8	21	3	4	0.389	0.330
Orikaza et al. 2014	20	11	26	19	4	3	0.340	0.379
FEMALES								
Martini et al. 2006	140	117	104	125	25	29	0.286	0.338
Tregouet et al. 2009	94	394	67	378	21	86	0.299	0.321
Steinbrugger et al. 2010	82	82	54	90	15	27	0.278	0.362
Antoni et al. 2011	476	354	421	334	123	74	0.327	0.316
Heit et al. 2012	365	250	312	345	81	74	0.313	0.368
de Hann et al. 2012	704	1140	634	1043	149	249	0.313	0.317
Tokgoz et al. 2012	21	21	15	27	5	8	0.305	0.384
Orikaza et al. 2014	63	51	73	46	14	13	0.303	0.327

Sensitivity analyses—which included the exclusion of one study that did not show consistency with HWE in our analysis, subgrouping by geographical region, high-risk group and proxy SNPs—did not modify the observed between-study heterogeneity (data not shown). We used geographic region for subgroup analyses as a proxy approach to assess putative effects due to ethnic heterogeneity. Secondary analyses of alternative genetic models showed that results from the allelic (A vs. G) and heterozygote (GA vs. GG) models were fairly consistent with those of the dominant model, where significant associations with thrombotic events were observed in females but not in males (Table 2). The strongest association was observed in the heterozygote model (despite smaller effective sample size), which is consistent with the previously described heterozygote advantage [30]. Based on the Venice criteria, there is moderate amount of evidence for association between Ala147Thr and thrombotic events in females.

**Table 2:** Sex specific meta-analysis with primary and secondary genetic models of the association between Ala147Thr variant and risk of venous thrombotic events.

Genotype Contrasts	Number of Studies	OR	95% CI	p-value	I^2^ (%)	P-value for Cochran’s Q
**Females (Number of cases = 4058 and Number of non-cases = 5367)**						
Allelic model	8	0.89	(0.80, 0.99)	0.041	54.7	0.031
GA vs. GG	8	0.80	(0.67, 0.96)	0.015	65.9	0.005
AA vs. GG	8	0.92	(0.78, 1.09)	0.325	11.6	0.340
**AA + GA vs. GG**	**8**	**0.81**	**(0.68, 0.97)**	**0.018**	**66.2**	**0.004**
AA vs. GA + GG	8	1.01	(0.88, 1.16)	0.885	0.0	0.507
**Males (Number of cases = 3222 and Number of non-cases = 3989)**						
Allelic model	8	1.03	(0.96, 1.11)	0.376	0.0	0.623
GA vs. GG	8	1.06	(0.96, 1.17)	0.248	0.0	0.874
AA vs. GG	8	1.06	(0.86, 1.31)	0.605	18.8	0.281
**AA + GA vs. GG**	**8**	**1.06**	**(0.96, 1.16)**	**0.263**	**0.0**	**0.263**
AA vs. GA + GG	8	1.03	(0.83, 1.12)	0.784	26.2	0.219

The primary genetic model is bolded and there was a statistically significant interaction between sexes (p = 0.004).

### Thr325Ile variant

In total, the meta-analysis included 15 studies that examined the association between the Thr325Ile variant and venous thrombosis (5347 cases and 8458 non-cases). The genotype distribution of Thr325Ile variant in case and non-case groups are shown in Table 3. The minor allele frequency in non-cases varied from 24.8% in Brazilian population [54] to 53.8% in Chinese population [45]. The only study that showed inconsistency with HWE was conducted in a non-European population [54].

**Table 3:** Distribution of *CPB2* Thr325Ile genotypes among cases and non-cases in studies included in the meta-analysis.

Included Study	CC Genotype		CT Genotype		TT Genotype		Minor Allele Frequency	
	Cases	Noncases	Cases	Noncases	Cases	Noncases	Cases	Noncases
**Venous Thrombosis**								
Morange et al. 2001	80	86	54	63	11	17	0.262	0.292
Zee et al. 2005*	59	56	43	54	18	10	0.329	0.308
Le Cam-Duchez et al. 2006	62	25	55	22	10	6	0.295	0.321
Martini et al. 2006	219	215	218	212	34	45	0.304	0.32
De Bruijne et al. 2007	40	53	69	52	9	13	0.369	0.331
Verdu et al. 2008	67	33	50	51	14	16	0.298	0.415
Heylen et al. 2006	70	37	63	27	11	5	0.295	0.268
Tregouet et al. 2009	201	584	173	518	32	124	0.292	0.312
Hoekstra et al. 2010	43	46	43	41	11	12	0.335	0.328
Kozian et al. 2010	99	1592	86	1275	21	277	0.311	0.291
Antoni et al. 2011	745	517	643	464	152	128	0.307	0.325
Heit et al. 2012	786	759	576	591	141	109	0.285	0.277
Li et al. 2012	27	15	42	44	11	21	0.4	0.538
Tokgoz et al. 2012	21	45	30	43	8	12	0.39	0.335
Orikaza et al. 2014	107	87	76	41	17	15	0.275	0.248

* Genotype counts calculated based on genotype and allele frequencies.

**Fig 3:**
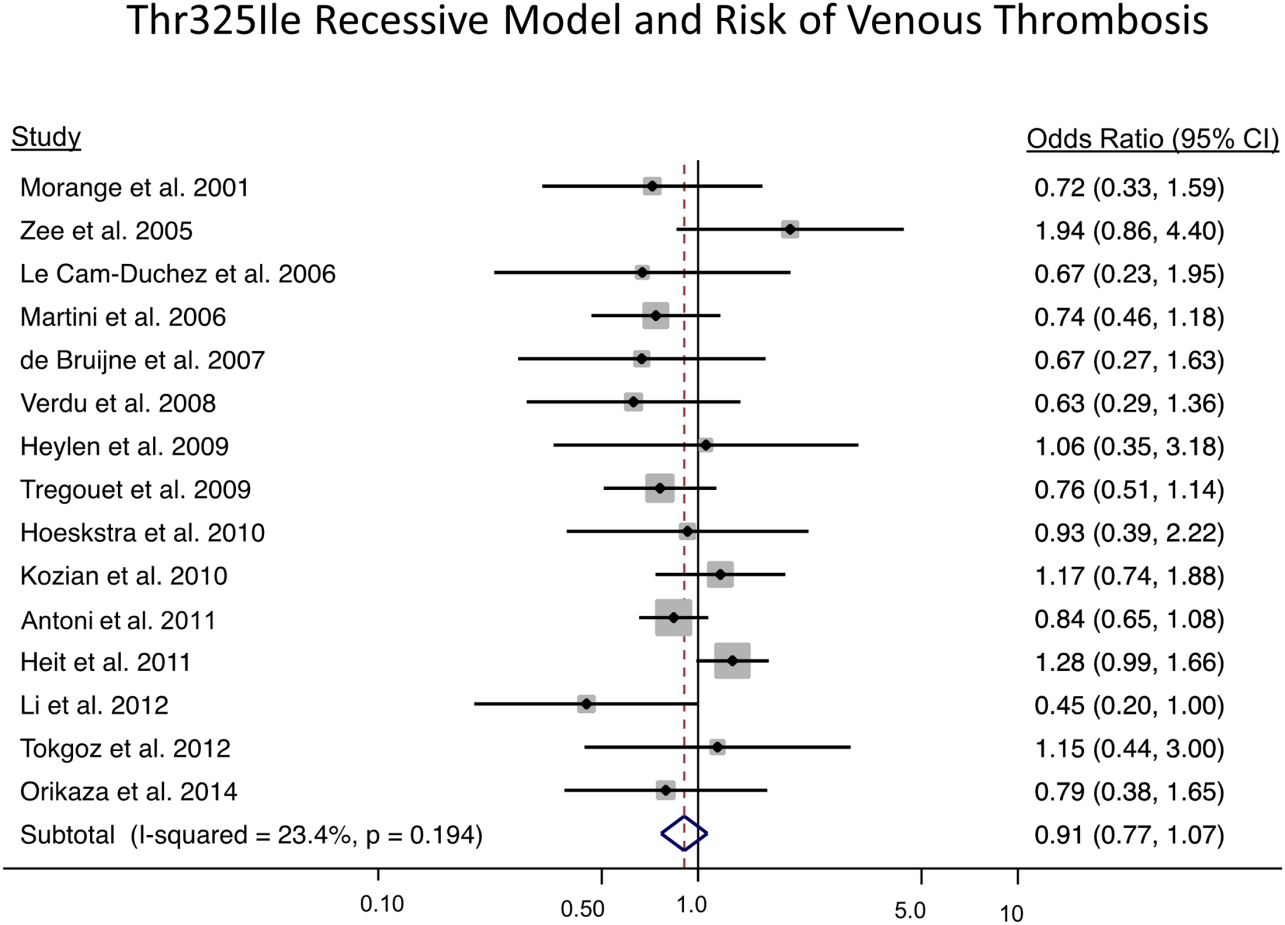
Meta-analysis results of the *CPB2* Thr325Ile variant and risk of venous thrombosis using a recessive model. The solid squares represent the ORs from the individual studies; horizontal lines represent corresponding CIs; the diamonds show the combined ORs.

Under the primary recessive model (TT vs. CT + CC), the odds of a venous thrombosis is 0.91 (95% CI: 0.77, 1.07; p = 0.244) (Fig 3). Overall, there was a low amount of heterogeneity (I^2^ = 23.4%, p = 0.194). There was no indication of publication bias (Egger’s test p = 0.330 and S3 Fig), or differences in allele frequencies by sex (p = 0.498). For the meta-analysis stratified by sex, 2416 female cases and 2823 female non-cases were analyzed from the six studies with sex specific data; and for males, 1873 cases and 1806 non-cases were included in the analysis from the seven studies with sex-specific data. Under a recessive genetic model, the interaction term to test differences between sexes was not significant (p = 0.665) where the OR in females was 0.99 (95% CI: 0.80, 1.232) and 0.93 (95% CI: 0.69, 1.24) in males. The Thr325Ile variant did not exhibit a sex-specific association.

**Fig 4:**
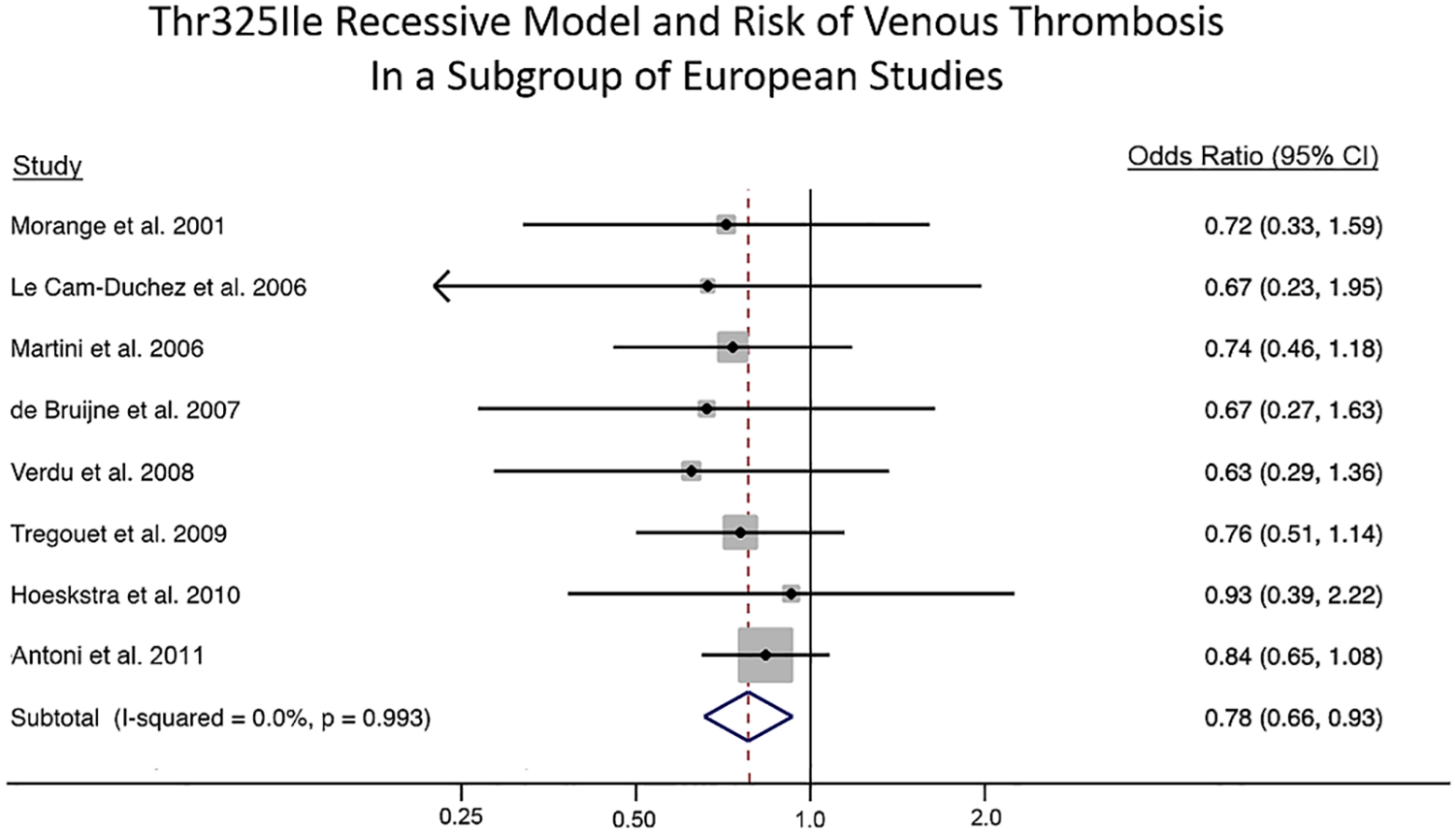
Meta-analysis results of the *CPB2* Thr325Ile variant and risk of venous thrombosis in European study populations. The solid squares represent the ORs from the individual studies; horizontal lines represent corresponding CIs; the diamonds show the combined ORs.

An examination of allele frequencies showed large variation across studies. As ethnicity was not clearly defined for each study, we used the study geographic region as a proxy. The subgroup analysis by geographic region showed odds of 0.83 (95% CI: 0.71, 0.97; p = 0.021) for venous thrombosis among the ten studies conducted in European countries (Fig 4). The remaining geographical regions had a limited number of studies: two in the United States, one in Africa, one in South America, and one in Asia, and were therefore not meta-analyzed. Exclusion of two studies on indication that the non-cases likely belonged to the case population (i.e. these individuals showed evidence of thrombotic events) or the case group included individuals predisposed to a thrombotic event reduced the odds for venous thrombosis to 0.78 (95% CI: 0.66, 0.93; p = 0.006) (S4 Fig). Restricting the analysis to high-risk study populations and excluding proxy SNPs did not affect the results (results not shown). The meta-analysis results under the primary and secondary genetic models in all European studies are presented in Table 4. Only recessive (TT vs. CT + CC) and homozygote (TT vs. CC) models showed significant associations for venous thrombotic events. Based on the Venice criteria there is moderate amount of evidence for association.

**Table 4:** Meta-analysis of the association between Thr325Ile variant and risk of venous thrombosis in European study populations.

Genotype Contrasts	Number of Studies	OR	95% CI	p-value	I^2^ (%)	P-value for Cochran’s Q
**Venous Thrombosis (Number of cases = 3235 and number of non-cases = 3486)**						
Allelic model	10	0.94	(0.87, 1.02)	0.139	11.5%	0.337
CT vs. CC	10	1.00	(0.88, 1.14)	0.994	22.6%	0.235
TT vs. CC	10	0.82	(0.70, 0.97)	0.021	0.0%	0.736
TT + CT vs. CC	10	0.97	(0.85, 1.10)	0.593	28.2%	0.185
**TT vs. CT + CC**	**10**	**0.83**	**(0.71, 0.97)**	**0.021**	**0.0%**	**0.923**

The primary genetic model is bolded and there was no interaction effect by sex.

## Discussion

Our strategy of implementing a systematic and comprehensive literature search, paired with persistent efforts to include unpublished studies and to collect sex-specific genotype counts on these variants by directly contacting authors permitted a thorough meta-analysis to be conducted. Applying biological, population genetics and evolutionary knowledge to select primary genetic models for candidate gene analysis allowed us to approach the data with *a priori* hypotheses and thus, avoid unnecessary multiple testing. Prior suggestions of sex differences in risk profiles for variants in the *CPB2* gene for cardiovascular disease [17] led to sex-specific analyses to detect associations that may otherwise be missed when both sexes are analyzed together.

### Ala147Thr variant

The dominant model was selected as the primary model for association as per evidence for a potential heterozygote advantage based on balancing selection in the exons 6–7 region of *CPB2* [30] and differences in TAFI levels for heterozygotes and homozygotes for the recessive allele [32, 60]. We showed that when the Ala147Thr variant is analyzed in males and females combined, there is no observed association with thrombotic events (OR = 0.94, p = 0.128). These results are consistent with previous results of a meta-analysis of this variant with coronary heart disease [34] but inconsistent with a recent meta-analysis with venous thrombosis [56], which reported an association across all genetic models.

There are key differences between the two venous thrombosis meta-analysis methodologies that may explain the discordant results. First, Qian et al. 2015 included much fewer studies than the current meta-analysis (e.g. 15 versus 8 studies for the Thr325Ile variant). Second, of the studies that were included by Qian et al., two studies appear to have overlapping study samples and included oncologic patients; differences in the etiologies of provoked (e.g. cancer) and idiopathic venous thrombosis are well documented [66], Third, several of the reported genotype counts for each genetic model do not match the genotype counts provided the in the original papers—it is unclear what the source of the discordance and consequently the effect estimates do not always match the unadjusted effect estimated provided in the original papers.

The analysis of males and females combined show higher heterogeneity between studies compared to males analyzed alone; however, significant heterogeneity still exists when the females were analyzed separately. The Ala147Thr variant showed a significantly higher minor allele frequency in females compared to males and was previously suggested to be associated with coronary heart disease in a sex-specific manner; together these justified the investigation of a sex-specific association. The results of our meta-analysis support a sex-specific association between Ala147Thr and all thrombotic events in females, where we observed 19% decreased risk with those having at least one copy of the minor allele, while no association is observed in males. This observation is consistent with the results of the single study by Silander et al. who showed that the Ala147Thr variant, under a dominant model, had a suggestive protective effect for coronary heart disease (hazard ratio (HR) = 0.31, 95% CI: 0.16, 0.60) in females but no effect in males (HR = 0.89, 95% CI 0.58–1.38) [17]. The size of the effect in the study by Silander et al is stronger than the effect observed in our meta-analysis, which may be due to the differences in study design and outcome (coronary artery disease versus venous thrombosis). Most of the studies included in our meta-analysis had a case-control design, while the study by Silander had a case-cohort design with time-at-risk data and an outcome of coronary heart disease, providing a more precise estimate of risk. Similarly, a study by Smith et al 2007, for which we did not have genotype information, observed a decreased risk for rs17844078, which is in high LD with Ala147Thr, in postmenopausal women but failed to replicate the association in a second study sample of both men and women [48]. Because individual studies did not examine a sex-specific effect, there is limited risk of publication bias for the sex-specific analysis.

### Thr325Ile variant

The recessive model was selected for the primary model based on evidence for homozygotes having changes in protein levels [31–33] and stability [26, 31]. Under a recessive model (TT vs. CT + CC), the meta-analysis OR for a venous thrombosis, across the 15 unique studies, was not overall significant. This result is inconsistent with the meta-analysis by Qian et al 2015, where they analyzed the association across eight studies and observed a significant association. As outlined above, there are a number of methodological differences; specifically study and sample size difference. The Thr325Ile variant showed allele frequency variation by geographical region. When the analysis was limited to the European population, we observed a statistically significant decreased risk of venous thrombosis (OR = 0.83, p = 0.021). The allele frequency of this variant has been demonstrated to vary between European and African populations [67]. In this meta-analysis, we also observed substantial variability by region. A previous meta-analysis examining Thr325Ile and coronary artery disease observed an overall increased risk (OR = 1.25, 95% CI: 1.02, 1.54) in mixed ethnicities, including: European, Asian, and African study populations [34]. However, in a subgroup analysis of only European studies, the association did not reach statistical significance (OR = 1.13, 95% CI: 0.90, 1.40) [34]. The opposing effect of the variant for venous thrombosis compared to coronary artery disease seems contradictory but it is biologically plausible. Venous thrombi are clots that are rich in fibrin and red blood cells. The 325-Ile variant is associated with lower plasma levels of TAFI [32, 33], and since TAFI works in a threshold dependent manner, there may not be adequate levels of TAFI to stabilize the clot, resulting in increased fibrinolysis. Arterial thrombi have a higher concentration of platelets because they are the result of a ruptured of atherosclerotic plaque, from having coronary artery disease. Platelets also secrete TAFI, which augment TAFI already present in plasma and enhance the anti-fibrinolytic effects [68], and therefore would act to stabilize the clot and increase the risk of arterial thrombosis. Therefore, it is possible for the opposing directions of association for venous compared to arterial thrombosis may be due to impaired or enhanced fibrinolytic activity. Additionally, TAFI is also involved in inflammation; changes in TAFI levels may result in abnormal regulation of inflammation [69] that is specific to arterial thrombosis, which may result in an increased risk for coronary heart disease.

### Strengths and limitations

*CPB2* Ala147Thr and Thr325Ile variants are the most widely studied variants in *CPB2* gene and have been previously associated with TAFI levels and activity. Both variants result in amino acid substitutions and offer biologically plausible mechanisms to alter the risk of thrombotic events. However, as for any genetic association studies, there is the potential that the associated variants are in fact in LD with other variants that are causally associated with disease risk. For instance the Ala147Thr is in LD with rs9526136 variant, which has been associated with intact TAFI and TAFI activation peptide [43]. Further, the study by Smith et al. 2007 identified a variant in the *CPB2* gene, rs17844078, that showed a similar association in females to the Ala147Thr variant; these two variants were in high LD [48], making it difficult to pinpoint the causal variant.

This systematic review included a comprehensive search of all articles assessing any *CPB2* variants and risk of a venous thrombotic event, and followed rigorous systematic review methodologies [58, 62, 63, 70]. A potential source of missing data is whether there was selective reporting of positive results. Tests for publication bias did not detect publication bias and many studies included reported on multiple variants and included null associations. Additionally, the inclusion of data from GWAS studies and unpublished results limit the susceptibility of this meta-analysis to publication bias. However, a meta-analysis is susceptible to the same potential sources of bias and error as the individual studies. Importantly, using genotype frequency to calculate crude OR and not adjusting for potential confounder or taking into account matching could potentially alter the results. The calculated OR in the meta-analysis corresponded to the adjusted OR presented in the individual studies, which suggest that bias due to known potential confounders is unlikely. Further, potential sources of bias were also assessed through several carefully conducted sensitivity analyses outlined in the methods.

## Conclusions

This meta-analysis provides evidence that the Thr325Ile variant under the recessive model is significantly associated with decreased risk of venous thrombosis in Europeans. Additionally, there is evidence to support a sex-specific association between the Ala147Thr variant and risk venous thrombosis in females under a dominant model. It is estimated that 15% of SNPs may act in a sex-dependent manner [16] and may help explain sexual dimorphism seen in the occurrence of thrombotic events. Although, most individual studies were unable to detect an association between *CPB2* variants and the risk of thrombotic events, this meta-analysis supports the role of *CPB2* variants in the risk of venous thrombosis.

## Supporting information

S1 Table Quality assessment items, with each item out of 2, adapted from the STREGA and HuGENet guidelines. (DOCX)

S2 Table Characteristics of studies included in the meta-analysis of either Thr325Ile and/or Ala147Thr variants and risk of venous thrombosis. (DOCX)

S3 Table Distribution of *CPB2* Ala147Thr genotypes among cases and non-cases in studies included in the meta-analysis. (DOCX)

S1 Fig Meta-analysis results of the *CPB2* Ala147Thr variant and risk of venous thrombosis in all studies. The solid squares represent the ORs from individual studies; horizontal lines represent corresponding CIs; the diamonds show the combined ORs.
(TIF)

S2 Fig Funnel plot was used to assess publication bias for all studies with genotype data on the Ala147Thr variant with the dominant model. (TIF)

S3 Fig Funnel plot was used to assess publication bias for all studies with genotype data on the Thr325Ile variant with the recessive model. (TIF)

S4 Fig Meta-analysis results of the *CPB2* Thr325Ile variant and risk of venous thrombosis in European study populations (excluding three studies). The solid squares represent the ORs from individual studies; horizontal lines represent corresponding CIs; the diamonds show the combined ORs.
(TIF)

S1 File Meta-analysis on genetic association studies checklist. (DOCX)
