# Supplementary material for: Sex-specific effect of CPB2 Ala147Thr but not Thr325Ile variants on the risk of venous thrombosis: A comprehensive meta-analysis
Source: PLoS One. 2017 May 26;12(5):e0177768. doi: 10.1371/journal.pone.0177768 (PMC5446132; doi:10.1371/journal.pone.0177768)
Supplement: S1 Table — (DOCX) [file pone.0177768.s001.docx]

| Item Number | Item | Category |
| --- | --- | --- |
| 1 | What was the study size? And was it large enough? | Research Methods (2) |
| 2 | Was the study design suitable? | Research Methods (2) |
| 3 | Selection of cases | Study Participants (2) |
| 4 | Selection of non-cases | Study Participants (2) |
| 5 | Was the genotyping success rate greater than 95%? | Genotype Information (1) |
| 6 | Were confounding factors accounted for? | Research Methods (2) |
| 7 | Was population stratification addressed? | Research Methods (2) |
| 8 | Was Hardy-Weinberg Equilibrium calculated and reported? Did it agree with the recalculation? | Genotype Information (1) |
