# Supplementary material for: Sex-specific effect of CPB2 Ala147Thr but not Thr325Ile variants on the risk of venous thrombosis: A comprehensive meta-analysis
Source: PLoS One. 2017 May 26;12(5):e0177768. doi: 10.1371/journal.pone.0177768 (PMC5446132; doi:10.1371/journal.pone.0177768)
Supplement: S2 Table — (DOCX) [file pone.0177768.s002.docx]

| Description | | | | Cases | | | | | Non-cases | | | |
| --- | --- | --- | --- | --- | --- | --- | --- | --- | --- | --- | --- | --- |
| Study | Design | Country or Region | Ethnicity or Race | N | Mean Age | % F | Ascertainment | Case definition | N | Mean Age | % F | Ascertainment |
|  |  |  |  |  |  |  |  |  |  |  |  |  |
| de Bruijne et al. 2007 [36] | Case-control | Netherlands | Caucasian | 118 | 51 | 54 | Patients presenting from seven hospitals | Objectively diagnosed Budd-Chiari syndrome or portal vein thrombosis | 118 | 51 (M) | 54 (M) | Partners or friends of the patient who had no history of thrombotic events |
|  |  |  |  |  |  |  |  |  |  |  |  |  |
|  |  |  |  |  |  |  |  |  |  |  |  |  |
| de Haan et al. 2012 [37] | Case-control (MEGA) | Netherlands | Caucasian | 2712 | 47 | 54.5 | Consecutive patients 18-70 years of age from 6 anticoagulation clinics | Objectively diagnosed first VTE | 4634 | 48 | 50.4 | Partner of patients and randomly selected controls through double digit dialling from same geographical region. |
| Antoni et al. 2011 [65] | Case-control (MARTHA) | France | Caucasian | 1542 | 44 | 70 | Consecutive patients recruited from the Thrombophilia center of La Timone hospital | Objectively diagnosed VTE without strong known risk factors | 1110 | 47 | 52 | Healthy population-based with not history of CVD and healthy heterozygotes for F5L, F2 G20210A mutations selected from national health examination centers. |
| Heit et al. 2012 [64] | Case-control | United States | Majority Caucasian | 1503 | 54.7 | 50.5 | Consecutive Mayo Clinic outpatients aged 18 years or older. | Objectively-diagnosed deep vein thrombosis or pulmonary embolism | 1459 | 55.5 | 52.4 | Prospectively select clinic-based controls from persons undergoing general medical examinations, matched on age, sex, state of residence, MI/Stroke status. |
| Heylen et al. 2009 [39] | Case-control | Belgium/Serbia | Caucasian | 144 | 36.5 | 68 | From a cohort study that selected consecutive patients with a history of VT or unexplained late pregnancy loss and carriers of inherited thrombophilia | Confirmed thrombosis event in symptomatic patients (121) and (23) carriers who were asymptomatic | 69 | 34 | 69.6 | Recruited among spouses of carriers, staff and students and no history of venous and arterial thrombosis |
| Hoekstra et al. 2010 [40] | Case-control (En-vie) | Europe | Majority Caucasian | 101 | 37 | 58 | Consecutive patients from 9 countries | Objectively diagnosed splanchnic vein thrombosis (Budd-Chiari syndrome and portal vein thrombosis) | 101 | M | M | Non-relatives of patients, matched for sex, ethnicity and age (3yrs) |
|  |  |  |  |  |  |  |  |  |  |  |  |  |
| Kozian et al. 2010 [42] | Cohort (LURIC) | Germany | Caucasian | Multiple Outcomes |  |  | Cohort of patients admitted to hospital for angina and underwent coronary angiography | Multiple outcomes, history of VTE, Stroke, MI based on patient records, and questionnaire data | 3358 | 62.6 | 29.6 | Cohort of patients admitted to hospital and underwent coronary angiography |
| Le Cam-Duchez et al. 2006 [44] | Cohort  (Pro-care subset) | France | Caucasian | 127 | 45 | 66.9 | Consecutive Pro-care cohort patients that were FV Leiden homozygotes | Symptomatic cohort patients, had a history of VTE | 53 | 36 | 77.4 | Asymptomatic Pro-care cohort patients that were FV Leiden homozygotes |
|  |  |  |  |  |  |  |  |  |  |  |  |  |
| Li et al. 2012 [45] | Case-control | China | Chinese | 80 | 54 (median) | 45 | Patients from Second Hospital of the University of West Branch and the mountain provinces of Shaanxi People's hospital | Objectively diagnosed DVT or PE | 80 | 55 (median) | 43.75 | Healthy individuals |
| Martini et al. 2006 [32] | Case-control (LETS) | Netherlands | Caucasian | 471 | 45 | 43 | Consecutive patients between 18 -70 years of age from thrombophilia centers | Objectively confirmed first DVT with no malignancy | 472 | 44.7 (M) | 43 (M) | Patient found an unrelated, healthy control of same age, and no history of VTE or cancer |
|  |  |  |  |  |  |  |  |  |  |  |  |  |
|  |  |  |  |  |  |  |  |  |  |  |  |  |
| Morange et al. 2001 [47] | Families | France | Caucasian | 155 | 47 | 59.2 | Consecutive heterozygous FV Leiden carriers from 220 unrelated families | Objectively confirmed VT | 145 | 35 | 57.9 | Asymptomatic relatives of carriers or individuals with family history or VT |
| Orikaza et al. 2014 [54] | Case-control | Brazil | Brazilian | 200 | 38.5 | 73 | Consecutive CVT and VTE outpatients >18 years of age from an anticoagulation clinic | Objectively diagnosed CVT or VTE | 143 | 37  (M) | 77  (M) | Acquaintances or partners of patients with no history of thromboembolic events |
| Steinbrugger et al. 2010 [49] | Case-control | Austria | Caucasian | 284 | 67.3 | 53.2 | Hospital based cases from the ophthalmology department | Objectively diagnosed central retinal vein occlusion | 335 | 69.1 | 59.4 | Hospital-based controls in the ophthalmology department with other conditions |
| Tokgoz et al. 2012 [55] | Case-control | Turkey | Turkish and Anatolian | 59 | 38 | 69.5 | Patients from the Neurology Department of Meram Faulty of Medicine | Objectively diagnosed adults (>18 years) with non-traumatic cerebral venous infraction | 100 | 28 | 60 | Chosen randomly from the population with no history of vascular disease |
| Tregouet et al. 2009 [50] | Case-control (EoVT) | France | Caucasian | 411 | 36 | 45 | Recruited from four French centers over 7 years | First objectively diagnosed, idiopathic VTE before 50 years of age | 1228 | 50 | 70 | Healthy, middle-aged, population-based volunteers from a clinical trial on antioxidant supplementation |
| Verdu et al. 2008 [51] | Case-control | Spain | Caucasian | 131 |  |  | Consecutive patients from one hospital, adults between 18 and 80 years of age | Objectively diagnosed DVT or PE | 100 | M | M | Individuals matched for age, sex, and race, who had no history of DVT or arterial disease |
| Zee et al. 2005 [53] | Nested Case-control (PHS) | United States | Majority Caucasian | 120 | 59 | 0 | Cohort of physicians 40 to 84 years of age | Objectively diagnosed idiopathic DVT or PE | 120 | 58.8 | 0 | Controls matched by age, smoking history, and time of randomization |
