# Supplementary material for: Sex-specific effect of CPB2 Ala147Thr but not Thr325Ile variants on the risk of venous thrombosis: A comprehensive meta-analysis
Source: PLoS One. 2017 May 26;12(5):e0177768. doi: 10.1371/journal.pone.0177768 (PMC5446132; doi:10.1371/journal.pone.0177768)
Supplement: S3 Table — (DOCX) [file pone.0177768.s003.docx]

| Included Study | | GG Genotype | | GA Genotype | | AA Genotype | | Minor Allele Frequency | |
| --- | --- | --- | --- | --- | --- | --- | --- | --- | --- |
|  |  | Cases | Noncases | Cases | Noncases | Cases | Noncases | Cases | Noncases |
| **Venous Thrombosis** | |  |  |  |  |  |  |  |  |
|  | Morange et al. 2001 | 100 | 78 | 64 | 63 | 4 | 4 | 0.214 | 0.245 |
|  | Le Cam-Duchez et al. 2006 | 79 | 22 | 45 | 25 | 12 | 6 | 0.254 | 0.349 |
|  | Martini et al. 2006 | 236 | 211 | 198 | 210 | 37 | 51 | 0.289 | 0.331 |
|  | de Bruijne et al. 2007 | 58 | 48 | 54 | 50 | 5 | 18 | 0.274 | 0.371 |
|  | Verdu et al. 2008 | 41 | 22 | 41 | 22 | 24 | 20 | 0.420 | 0.484 |
|  | Tregouet et al. 2009 | 199 | 579 | 168 | 528 | 44 | 121 | 0.311 | 0.314 |
|  | Kozian et al. 2010 | 92 | 1388 | 74 | 1193 | 23 | 287 | 0.317 | 0.308 |
|  | Steinbrugger et al. 2010 | 149 | 147 | 107 | 152 | 28 | 36 | 0.287 | 0.334 |
|  | Antoni et al. 2011 | 710 | 528 | 659 | 477 | 173 | 105 | 0.326 | 0.309 |
|  | de Hann et al. 2012 | 1323 | 2160 | 1218 | 1968 | 269 | 471 | 0.312 | 0.316 |
|  | Heit et al. 2012 | 697 | 672 | 635 | 650 | 171 | 137 | 0.325 | 0.317 |
|  | Li et al. 2012 | 24 | 28 | 47 | 36 | 9 | 16 | 0.406 | 0.425 |
|  | Tokgoz et al. 2012 | 28 | 40 | 23 | 48 | 8 | 12 | 0.331 | 0.360 |
|  | Orikaza et al. 2014 | 83 | 62 | 99 | 65 | 18 | 16 | 0.338 | 0.339 |
