# Supplementary figures and images for: Sex-specific effect of CPB2 Ala147Thr but not Thr325Ile variants on the risk of venous thrombosis: A comprehensive meta-analysis
Source: PLoS One. 2017 May 26;12(5):e0177768. doi: 10.1371/journal.pone.0177768 (PMC5446132; doi:10.1371/journal.pone.0177768)

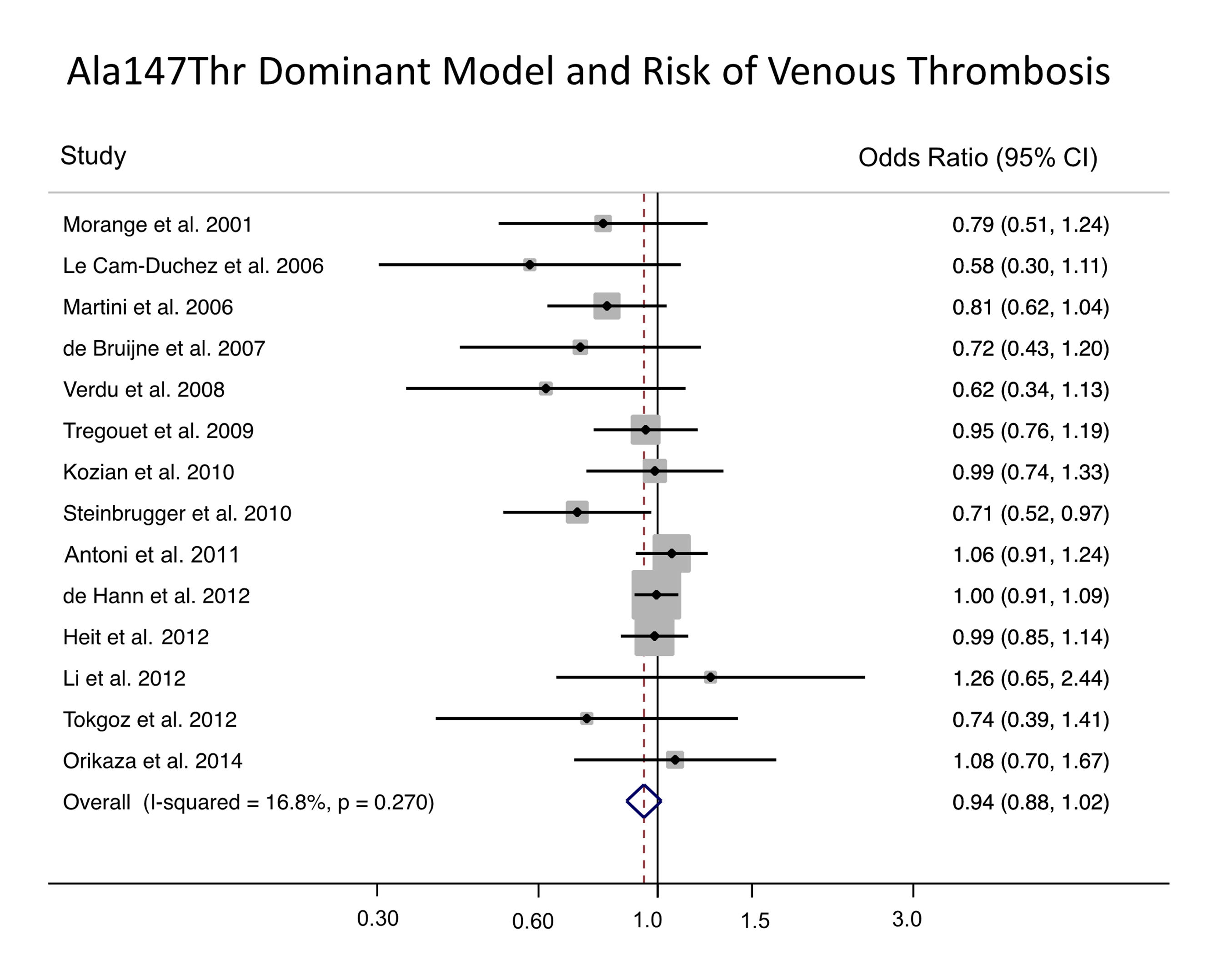

Supplement: S1 Fig — The solid squares represent the ORs from individual studies; horizontal lines represent corresponding CIs; the diamonds show the combined ORs. (TIF) [file pone.0177768.s004.tif]

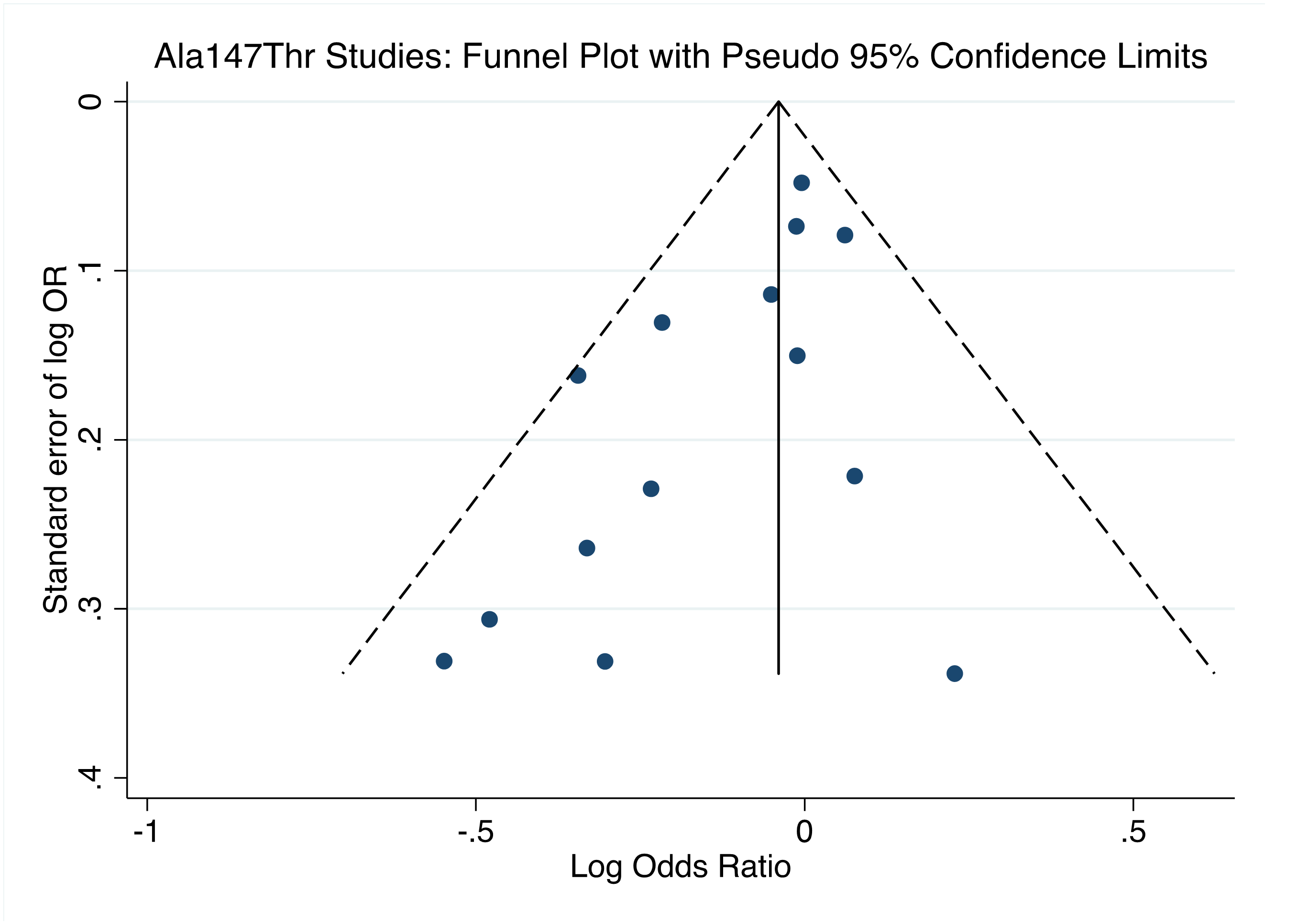

Supplement: S2 Fig — (TIF) [file pone.0177768.s005.tif]

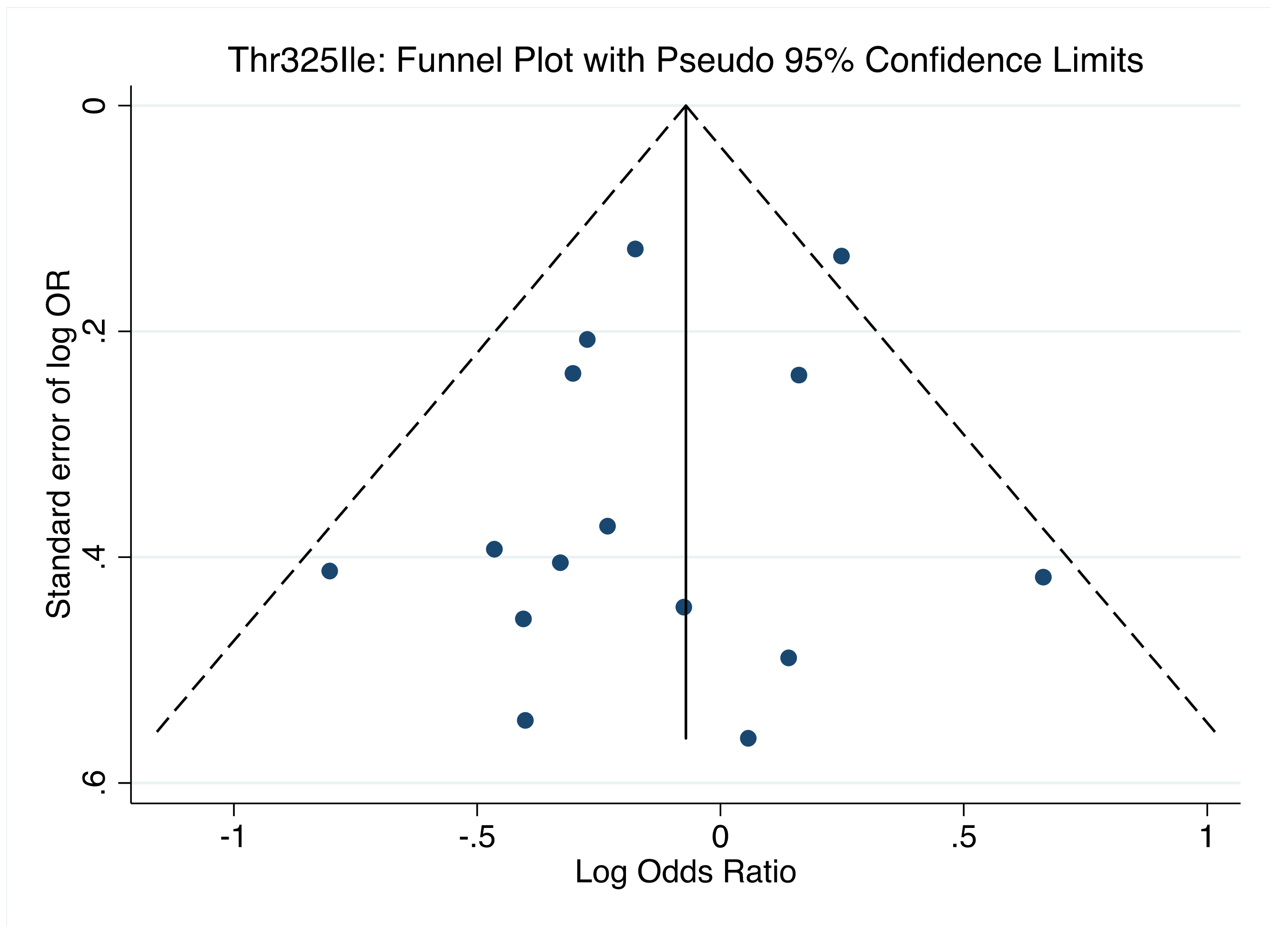

Supplement: S3 Fig — (TIF) [file pone.0177768.s006.tif]

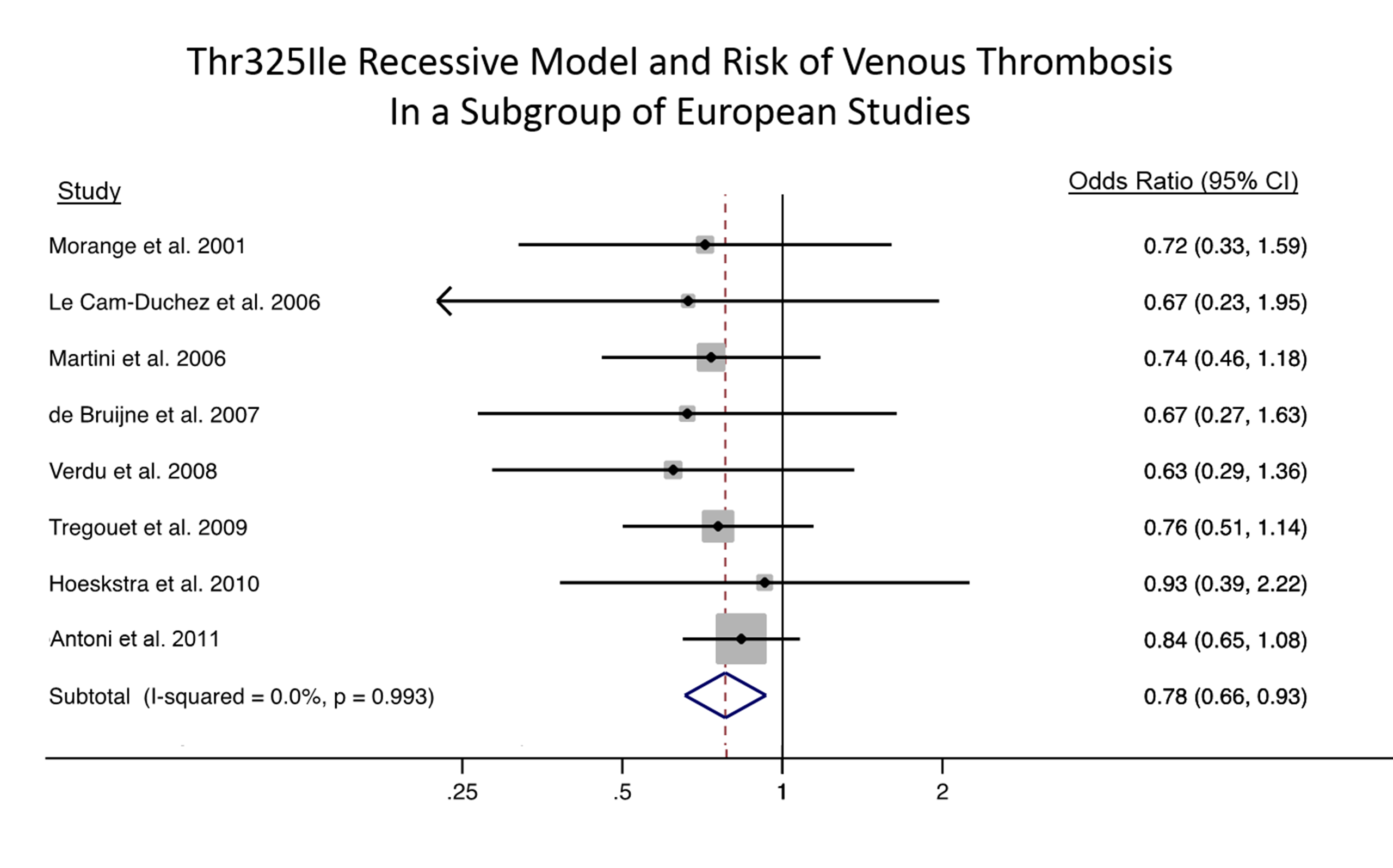

Supplement: S4 Fig — The solid squares represent the ORs from individual studies; horizontal lines represent corresponding CIs; the diamonds show the combined ORs. (TIF) [file pone.0177768.s007.tif]
